# Spinal Plasticity and Behavior: BDNF-Induced Neuromodulation in Uninjured and Injured Spinal Cord

**DOI:** 10.1155/2016/9857201

**Published:** 2016-09-19

**Authors:** Sandra M. Garraway, J. Russell Huie

**Affiliations:** ^1^Department of Physiology, Emory University School of Medicine, 615 Michael Street, Atlanta, GA 30307, USA; ^2^Brain and Spinal Injury Center, University of California, San Francisco, 1001 Potrero Avenue, San Francisco, CA 94110, USA

## Abstract

Brain-derived neurotrophic factor (BDNF) is a member of the neurotrophic factor family of signaling molecules. Since its discovery over three decades ago, BDNF has been identified as an important regulator of neuronal development, synaptic transmission, and cellular and synaptic plasticity and has been shown to function in the formation and maintenance of certain forms of memory. Neural plasticity that underlies learning and memory in the hippocampus shares distinct characteristics with spinal cord nociceptive plasticity. Research examining the role BDNF plays in spinal nociception and pain overwhelmingly suggests that BDNF promotes pronociceptive effects. BDNF induces synaptic facilitation and engages central sensitization-like mechanisms. Also, peripheral injury-induced neuropathic pain is often accompanied with increased spinal expression of BDNF. Research has extended to examine how spinal cord injury (SCI) influences BDNF plasticity and the effects BDNF has on sensory and motor functions after SCI. Functional recovery and adaptive plasticity after SCI are typically associated with upregulation of BDNF. Although neuropathic pain is a common consequence of SCI, the relation between BDNF and pain after SCI remains elusive. This article reviews recent literature and discusses the diverse actions of BDNF. We also highlight similarities and differences in BDNF-induced nociceptive plasticity in naïve and SCI conditions.

## 1. Introduction 

**Figure 1:**
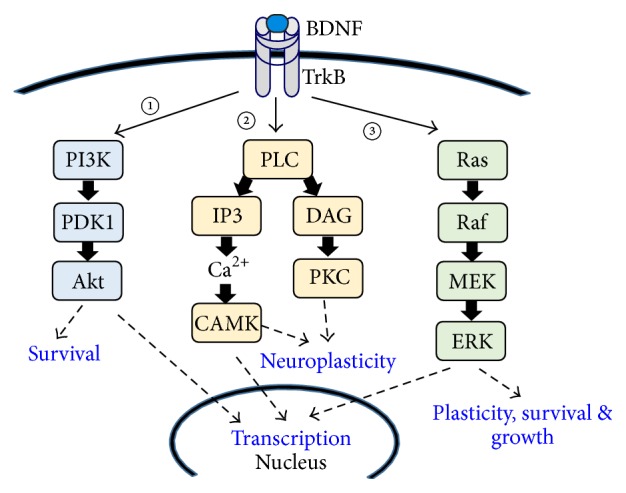
BDNF-TrkB dependent pathways: BDNF binds its high affinity receptor Tropomyosin-related kinase B (TrkB) and activates three main pathways (adapted from [3–5]): ① the PI3K/Akt pathway which has been shown to mediate cell survival function of BDNF, ② the phospholipase C (PLC) pathway which leads to the activation of protein kinase C (PKC), and ③ the Ras pathway which activates extracellular signal-regulated kinase (ERK). Activation of the kinases, PKC and ERK, leads to posttranslational modification such as phosphorylation and transcription. These processes are critical to neuroplasticity, including central sensitization and LTP. BDNF also binds the nonselective p75^NTR^.

After three decades of research, significant advances have been made in unraveling the cellular effects of brain-derived neurotrophic factor (BDNF). BDNF is a member of the neurotrophin family of growth factors that is encoded by the* bdnf *gene. BDNF was purified as the second member of the neurotrophin family in 1982 by Barde et al. [1]. Mature BDNF (14 kD) is cleaved from pro-BDNF by a series of serine proteases or convertase enzymes such as furin, PACE4, and PC5/6-B [2]. The serine protease tissue plasminogen activator has also been shown to play a role in the cleavage of pro-BDNF into mature BDNF. Mature BDNF exists as a dimer and mediates its cellular functions through two receptors: the high affinity, ligand-specific tropomyosin receptor kinase B (TrkB) and the p75 neurotrophin receptor (p75^NTR^), which is a low affinity, nonselective neurotrophic receptor. Upon BDNF binding the TrkB receptor, a number of events follow. (i) The TrkB receptor dimerizes, which leads to (ii) autophosphorylation of the receptor and (iii) the subsequent activation of intracellular signaling cascades. These include the mitogen-activated protein kinase (MAPK), phospholipase C-*γ* (PLC-*γ*), and phosphatidylinositol-3 kinase (PI3-K) cascades (see [3–7] and Figure 1). Activation of these pathways leads to a myriad of cellular actions including synaptic modulation and neuroplasticity, cell survival, axonal elongation, and neurite outgrowth. The activation of p75^NTR^ similarly produces a variety of cellular events, ranging from neuronal differentiation to apoptotic cell death. Numerous studies have shown that p75^NTR^ is the preferred receptor via which the precursor protein, pro-BDNF, mediates various cellular actions.

Neural plasticity is essential to physiological functions. BDNF is a potent modulator of neural plasticity. It exerts diverse modulatory actions that range from neurogenesis to learning and memory formation. BDNF's actions appear synonymous with neuromodulation. While BDNF does not necessarily initiate an event such as a synaptic, sensory, or motor response, it can modify the response, exerting inhibition or facilitation. Such effects have been observed in the dorsal root ganglia, spinal cord, and various brain regions, where BDNF actions are typically associated with increased excitability, pronociception, learning, and memory. An important investigation into BDNF's effects focuses on its ability to modify injury-induced plasticity. BDNF has been shown to mediate inflammatory and peripheral injury-induced pain. However, as discussed below, after spinal cord injury (SCI), BDNF's actions appear to be more complex. Whereas treatments that increase spinal levels of BDNF are shown to promote functional recovery and adaptive plasticity, their effects on sensory functions, nociception, and pain remain elusive.

It should not be surprising that BDNF also exerts deleterious effects, which are typically associated with its overexpression. Although much can be said about the variety of outcomes that arise from pro-BDNF and mature BDNF interactions with both TrkB and p75^NTR^, some of which we briefly discuss, in this article, our goal is to provide a comprehensive review on the neuromodulatory actions of BDNF, with greater emphasis on the effects of mature BDNF. We will (1) offer an overview of BDNF as a modulator of neural plasticity and (2) discuss the differential roles BDNF plays in spinal plasticity in intact and injured spinal cord, focusing on BDNF's effect on nociceptive plasticity. Despite the significant progress that has been made over the last 30 years, we will show that many questions remain unanswered.

## 2. BDNF Is an Important Modulator of Neural Plasticity

The heterogeneity of BDNF's actions is due to its ability to engage distinct signaling pathways (Figure 1). Early investigations into these signaling mechanisms showed that BDNF signaling is involved in the formation, maturation, and function of excitatory and inhibitory synapses [8–12] and in synaptic plasticity [12, 13].

### 2.1. Overview of Plasticity

Plasticity, “the ability to adapt,” is an important feature of the central nervous system (CNS) [14–16]. Neural and functional changes that occur during development and maturation are crucial examples of plasticity. BDNF functions as a modulator of plasticity during development and into adulthood. A well-established role of BDNF is its ability to promote neuronal survival and development. This important function was initially demonstrated in the visual system, for example, [17, 18] and extended to other sensory systems such as the vestibular-auditory system [19]. Earlier studies revealed that, within the CNS, BDNF promotes neuronal development, although several of these studies indicated a preference towards sensory neurons, for example, [20–22]. The study by Jones et al. [20] showed that targeted disruption of the BDNF gene reduced the survival of offspring past 2 days after birth. Moreover, in offspring that survived, brain and sensory neuron developments were severely reduced. Along with the overwhelming number of reports demonstrating a preferential role of BDNF in developing sensory systems, several studies have shown that BDNF similarly functions as a survival factor for developing motoneurons [23, 24]. It is clear from these studies that, during early developmental periods, BDNF promotes adaptive plasticity, although the mechanisms that drive these actions have not been fully elucidated.

The fundamental ability of the CNS to function during postdevelopment and into mature stages relies largely on plasticity of synaptic connections. This includes changes in the number and efficacy of synaptic connections, which can ultimately direct neuronal activity. In addition to functioning as a critical survival factor during development, BDNF exerts numerous actions in developing and mature neural systems which draw on its ability to mediate or modify activity-dependent synaptic plasticity, learning, and memory. In many brain regions, BDNF plays a role in synaptic plasticity, although this has been most studied in the hippocampus, a brain region that encodes memory and learning. Long-term potentiation (LTP) is defined as an activity-dependent maintained increase in synaptic efficacy [25]. LTP, which is presumably the best described form of synaptic plasticity, has been proposed as a neural substrate of learning and memory. In general, its induction and maintenance require various cellular substrates such as the NMDA receptor, signaling kinases, posttranslational modification (e.g., phosphorylation), and transcription.

### 2.2. BDNF's Role in LTP, Learning, and Memory

As a modulator of neural plasticity, studies show that BDNF is implicated in the induction and maintenance of LTP. Initial investigation showed that BDNF induces membrane depolarization and subsequent firing of action potentials in hippocampal neurons [26]. However, several additional studies reported a more direct role of BDNF in the induction of hippocampal LTP. For instance, exogenous administration of BDNF produces synaptic facilitation [27, 28]. Several studies using pharmacological or genetic approaches show that the synaptic facilitation is mediated primarily via the TrkB receptor. For example, administration of the fusion protein TrkB-IgG, which scavenges endogenous BDNF, attenuated the synaptic response induced by tetanic stimulation and the subsequent LTP [27]. BDNF similarly plays a role in the late phase of LTP [29], a process requiring the induction of gene transcription [30]. Late phase LTP is significantly attenuated when endogenous BDNF is sequestered with TrkB-IgG [31]. Genetic approaches have also been used to substantiate the importance of BDNF signaling via the TrkB receptor in the development of LTP. Minichiello et al. [32] showed that LTP in CA1 is severely impaired following conditional knockout of the TrkB receptor in the forebrain. In recent years, studies have continued to describe possible mechanisms by which BDNF contributes to hippocampal LTP. These include a recent study by Leal et al. [33] wherein it was reported that hippocampal plasticity, evidenced as an increase in synaptic activity, is accompanied by BDNF-mediated trafficking of ribonucleoproteins to dendrites. Similarly, Edelmann et al. [34] showed that, in the hippocampus, timing-dependent LTP induced by repeated pairing of one presynaptic action potential with four postsynaptic spikes requires BDNF/TrkB signaling and insertion of new AMPA receptors. Notably, these studies demonstrate how essential BDNF is to hippocampal LTP.

As previously stated, long-term maintenance of synaptic efficacy or LTP has long been identified as a possible neural mechanism of memory and learning. Since BDNF is necessary for hippocampal LTP and long-term maintenance of synaptic efficacy and/or LTP has been implicated as a mechanism of learning and memory, BDNF would be expected to affect behavioral manifestations of learning and memory* in vivo*. Several studies are consistent with this possibility. For example, in adult rats, exogenous administration of BDNF into the hippocampus protects from the development of stress-induced learning and memory impairment [35], and stress-induced impairment of learning and memory is marked by a decrease in hippocampal levels of BDNF mRNA [36]. Genetic, as well as pharmacological, approaches have also been instrumental in providing such evidence. Deficits in endogenous BDNF levels produce severe impairments in spatial learning and memory tasks in both mice [37] and rats [38]. The contribution BDNF makes to learning and memory extends to brain regions other than the hippocampus. For example, conditional knockout of TrkB receptors in the forebrain of mice resulted in impaired learning behaviors [32], and mice overexpressing TrkB receptors in the cortex and hippocampus showed facilitated learning [39]. A recent study by Ju et al. [40] reported that, in adult rats, BDNF signaling in the amygdala is necessary for conditioned place aversion (CPA) behavior induced by naloxone-precipitated morphine withdrawal. In this study, CPA was accompanied with elevated BDNF levels but was completely blocked by inhibition of BDNF in the amygdala. Many additional studies strongly implicate BDNF signaling through the TrkB receptor in learning and memory. Also, they typically illustrate a direct role of BDNF in both cellular/synaptic (e.g., LTP) and behavioral aspects of learning and memory.

### 2.3. Other “Deleterious” Effects of BDNF

The various actions of BDNF also include deleterious effects. Although not exclusively, these effects are more commonly linked to the overexpression of BDNF and p75^NTR^ signaling. The expression of inflammatory and neuropathic pain could easily be seen as an important maladaptive effect of BDNF. As we discuss below, the development of pain is intricately associated with an increase in the expression of BDNF. However, there are several additional effects of BDNF that are deleterious in nature, which we briefly discuss in this section. First, epileptogenesis has been linked to the overexpression of BDNF in the hippocampus and/or cortex [41]. Supporting a role of BDNF in the development of epilepsy, a study by Kokaia et al. [42] showed that epileptogenesis is markedly suppressed in mutant mice expressing reduced levels of BDNF. BDNF's effect on epileptogenesis might be mediated through its high affinity receptor, TrkB. Several studies have shown that manipulations that perturb BDNF-TrkB signaling reduce the development of epilepsy. In contrast, overexpression of the full-length TrkB receptor, but not the truncated TrkB receptor, promotes epileptogenesis [43]; also see [44]. Given that the imbalance of inhibition and excitation is crucial to the development of epilepsy, these observations show that BDNF exerts modulatory control over both inhibitory and excitatory synaptic transmission in the brain.

Apoptotic cell death is a second deleterious effect of BDNF signaling system. However, this effect appears to be mediated by p75^NTR^ which is not only a BDNF-specific receptor, but also activated by nerve growth factor (NGF), neurotrophin- (NT-) 3, and NT-4/NT-5. The role p75^NTR^ plays in apoptotic cell death has been well studied. In a study by Roux et al. [45], it was reported that, after pilocarpine-induced seizure, the expression p75^NTR^ is increased in neurons undergoing apoptosis. Studies from the Hempstead laboratory showed that p75^NTR^ plays a role in producing apoptotic death of both neuronal [46, 47] and nonneuronal [48] cells. Additionally, they provided evidence that p75^NTR^-mediated apoptosis can be initiated by pro-BDNF [46]. Mature BDNF activation of the p75^NTR^ has also been shown to induce developmental apoptosis in sympathetic neurons [49]. Whereas studies consistently show that pro-BDNF-p75^NTR^ signaling induces apoptosis (also see Koshimizu et al. [50]), it should be noted that this interaction results in neuronal remodeling [51] and shapes neurite outgrowth [52]. A study by Fan et al. [53] showed that pro-BDNF-p75^NTR^ signaling can lead to selective death of axotomized sensory neurons following sciatic nerve transection. Clearly, pro- and mature BDNF actions through p75^NTR^ signaling have important physiological and cellular functions.

BDNF is also implicated in the stress response. Glucocorticoid receptors can interact with the TrkB receptor to modulate BDNF signaling [54, 55]. Studies typically show that glucocorticoids or their receptor ligands [55, 56] and stress [57–59] can suppress BDNF levels or its downstream signaling (also see reviews by Jeanneteau and Chao [60] and by Suri and Vaidya [61]). More recent studies implicating BDNF in stress have shown epigenetic regulation of the BDNF gene in response to various stress paradigms [62–64]. BDNF also plays a role in the pathophysiology of depression presumably by interacting with the monoaminergic systems (see reviews [65–67]). There are several other effects of BDNF signaling mechanisms that can produce devastating consequences. However, in the following sections of this review, we aim to discuss the role BDNF plays in nociceptive plasticity and pain in the intact and injured spinal cord.

### 2.4. BDNF's Role in Spinal Plasticity

The concept that chronic pain is maintained as a “memory trace” was first put forward by Coderre and Melzack [68]. This led to studies aimed at identifying commonalities between the neural mechanisms that underlie pain and those that encode learning and memory processes. Furthermore, as central sensitization, which is an increase in the excitability of the neuronal networks in the central nervous system, is considered the most likely neural substrate underlying spinal nociception and pain [69], many studies focused on mapping the neural processes and specific mediators involved. Given the similarity that exists between LTP and central sensitization at the cellular level, BDNF was initially proposed to play a critical role in central sensitization and inflammatory pain in the late 1990's with studies originating from the McMahon and Thompson laboratories. The results from these earlier studies suggested that, under inflammatory conditions, BDNF levels are increased in sensory neurons and this increase causes a subsequent facilitation of nociceptive spinal reflex excitability [70]. This study by Kerr et al. [70] first identified BDNF as critical to the development of inflammatory pain. Additionally, it identified small diameter “pain” fibers as a primary source of spinal BDNF, particularly after peripherally induced inflammation. To further demonstrate that BDNF plays a role in pain, it also showed that inflammatory pain induced by administration of formalin or carrageenan is significantly attenuated by TrkB-IgG. Thus, these observations demonstrated a novel function of BDNF; that is, elevated spinal levels of endogenous BDNF are necessary for inflammatory pain behaviors. In addition to these behavioral results, BDNF actions consistent with nociception were also observed at the cellular level. Specifically, Thompson et al. [71] showed that exogenous application of BDNF enhanced C fiber evoked ventral root reflexes and pretreatment with TrkB-IgG attenuated the amplitude of the ventral root potentials. Since these initial studies were undertaken, numerous studies have examined the role BDNF plays in sensory plasticity: central sensitization, nociception, and pain in intact CNS. The general consensus from many of these studies is that BDNF typically elicits pronociceptive actions, ranging from an increase in neuronal excitability to mediating pain behaviors, for example, [72–75].

Several additional studies have successfully associated BDNF with the development of pain. BDNF is upregulated in sensory neurons after peripheral inflammation induced by NGF or complete Freund's adjuvant [76–78]. In addition, inflammatory pain may cause a phenotypic switch of BDNF-expressing neurons, so that even large diameter sensory neurons express BDNF [79]. Lever et al. [80] showed that BDNF is released into the dorsal horn following electrical stimulation of afferent fibers at C fiber strength or after chemical stimulation by capsaicin. These apparent nociceptive actions of BDNF are not confined to DRG-to-dorsal horn neurons. Instead, even at supraspinal centers, BDNF-TrkB signaling appears to mediate descending pain facilitation [81].

BDNF is also implicated in the development of chronic neuropathic pain. For instance, several studies have shown elevated BDNF levels in sensory neurons and the dorsal horn in a variety of chronic neuropathic pain models [82–86]. Also, as observed in inflammatory pain, a phenotypic switch wherein larger diameter sensory neurons express BDNF is observed following sciatic nerve lesion [87]. The observation that BDNF is upregulated in sensory neurons of BDNF in these primary afferent fibers is consistent with its role in promoting nociceptive plasticity and pain (also see review by Pezet and McMahon [88]).

**Figure 2:**
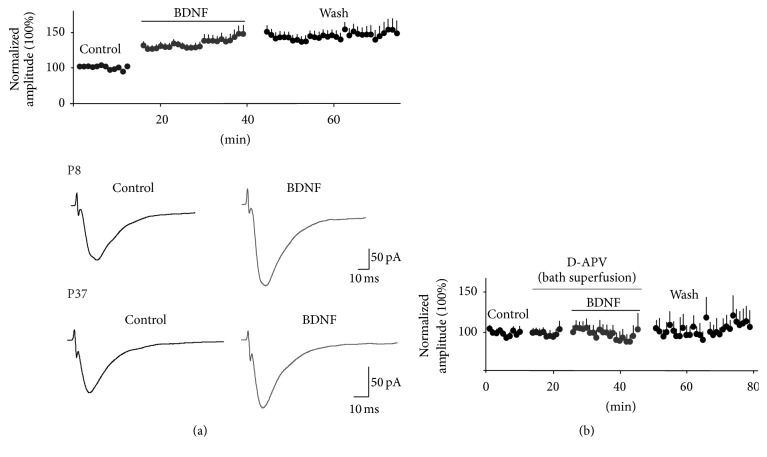
BDNF facilitates synaptic responses. (a) (Top) bath-applied BDNF potentiates dorsal root-evoked synaptic responses in lamina II neurons. In the presence of BDNF, synaptic amplitude is increased by ~30% and remains facilitated during wash. The data represent the average change in synaptic amplitude for all neurons, obtained from animals postnatal days 1–14. The average synaptic amplitude before BDNF application was computed for each neuron and was then normalized to the mean baseline amplitude denoted as 100%. The mean percent change in synaptic amplitude was calculated as the difference between the mean peak amplitude during drug treatment or wash and the mean value of the synaptic responses before drug (control). (a) (Bottom) the average synaptic responses are shown for recordings obtained in P8 and P37 rats before and during BDNF. Note: BDNF-induced facilitation is observed at both ages. (b) Bath application of the NMDA receptor antagonist, D-APV for 10 min prior to and during BDNF application blocks BDNF-induced synaptic facilitation (*n* = 14). Data previously reported in Garraway et al. [89, 90].

**Figure 3:**
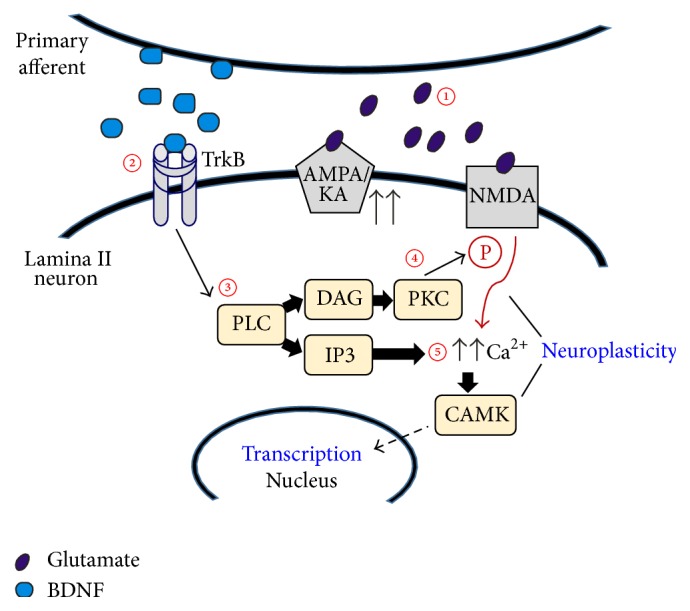
Proposed mechanism of BDNF-induced synaptic facilitation. Small diameter primary afferents express glutamate and BDNF. ① Under normal conditions, dorsal root stimulation evokes stable glutamatergic synaptic responses in lamina II neurons. ② Increased excitability of primary afferents causes the release of both glutamate and BDNF, which binds to TrkB receptors. Engagement of the TrkB receptors recruits the ③ PLC which can lead to activation of ④ PKC and ⑤ an increase in intracellular calcium [Ca^2+^]_i_. Both PKC and calcium dependent kinases such as CAMK can phosphorylate glutamatergic receptors, thereby increasing their calcium permeability. Consequently, these processes lead to an NMDA-R dependent facilitation of glutamatergic currents. Adapted from data reported in Garraway et al. [89].

To investigate BDNF's actions as a mediator of nociceptive plasticity of spinal neuronal networks, in Mendell's laboratory, we performed electrophysiological experiments to assess BDNF's effects at the synaptic level [89]. We used a transverse spinal cord slice preparation in neonatal and young rats to study the effect bath-applied BDNF has on NMDA-induced currents and dorsal root-evoked synaptic responses in lumbar 2- (L2-) L5 lamina II neurons. After characterizing the synaptic responses as AMPA/kainate receptor mediated and elicited primarily by electrical stimulation of small diameter, high threshold afferents (C fibers), we found that BDNF produced a long-lasting (irreversible) potentiation of the synaptic responses. Bath-applied NMDA-induced inward currents were also facilitated by BDNF. We also showed that BDNF-induced facilitation of synaptic currents is blocked by NMDA receptor blockade with D-APV (Figure 2). A more thorough investigation into the underlying mechanisms of BDNF-induced synaptic facilitation revealed that postsynaptic NMDA receptors, postsynaptic PLC-*δ*-PKC signaling, and increases in intracellular calcium in the lamina II neuron are all required for BDNF facilitation. These observations, which are summarized in Figure 3, indicate the similarity in the underlying neural mechanism that produces central sensitization or nociception with that mediating BDNF-induced synaptic plasticity. Lamina II receives synaptic input primarily from C fibers and is the initial nociceptive processing site within the CNS. Therefore, our results suggested that BDNF's pronociceptive actions might arise from its ability to produce a maintained (LTP-like) synaptic modification of excitatory responses in lamina II neurons.

A prolonged facilitation of nociceptive synaptic responses in lamina II may represent one of several mechanisms that underlie BDNF's role in pain/nociception. Other investigators showed that an increase in BDNF from activated spinal microglia is critical to peripheral injury-induced pain [96–99]. Importantly, these observations were the first to show that activated microglia release BDNF. These studies also indicated that BDNF released from activated microglia could function as the critical signaling molecule that bridges glia and neuronal associations that underlie neuropathic pain. The study by Coull et al. [96] was groundbreaking on many fronts. It showed that neuropathic pain after nerve injury results from a BDNF-mediated shift in neuronal anion gradient, which causes the inhibitory neurotransmitter GABA to produce excitatory currents [96, 100]. These effects of BDNF result from the intricate interaction between BDNF and the chloride transporter, KCC2 (discussed below, also see Figure 7). In addition, for the first time there was evidence that resident spinal cells release BDNF. This assertion dispelled the previous dogma that small diameter primary afferents are the only source of spinal BDNF. Overall, the study reinforced the critical role BDNF and microglia play in injury-induced pain hypersensitivity [96].

### 2.5. BDNF-TrkB Signaling in Nociceptive Plasticity and Pain

As previously mentioned, BDNF binds the TrkB receptor with high affinity leading to the activation of the PI3K-Akt, PLC, and MAPK/ERK pathways. Many studies provided a direct evidence to support BDNF-TrkB signaling in the development of inflammatory and/or neuropathic pain. An earlier study by Mannion et al. [72] showed that peripheral inflammation and C fiber electrical activity that increased BDNF expression in the DRG also increased full-length TrkB receptor levels in the dorsal horn (also see [85, 101]). Even in the brainstem, TrkB levels are elevated in a model of chronic inflammatory pain [102].

The MAPK/ERK and PLC-*γ*-PKC pathways are shown to play a role in mediating the pronociceptive effects of BDNF. Activation of ERK and PKC pathways by BDNF can induce phosphorylation of the NMDA-R1 subunit [103]. Both activated ERK and phosphorylated NMDA-R1 are essential to the development of inflammatory pain [104, 105]. In our initial investigation into BDNF's actions on spinal pain systems, we showed that inhibition of PLC-*γ*-PKC signaling blocks BDNF-induced facilitation of C fiber evoked synaptic responses in lamina II [89]. Activation of the PLC-*γ* pathway leads to increases in intracellular calcium, either from internal calcium stores or via phosphorylated calcium-permeable glutamate receptors. Hence, to further support the importance of PLC-*γ* mediated pathways, our study also showed that BDNF-induced facilitation of synaptic and NMDA-induced currents were blocked when intracellular calcium in the spinal neurons was chelated with BAPTA [89] (Figure 3). These observations are consistent with the many reports showing that stimulation of the MAPK/ERK and PLC-*γ*-PKC, activation of critical downstream kinases, and elevated levels of intracellular calcium are crucial processes in the development of nociceptive plasticity, inflammatory, and neuropathic pain (Figure 8). They also suggest that BDNF can evoke pronociceptive actions by engaging these intracellular mechanisms, postsynaptically.

## 3. BDNF Impacts Plasticity after SCI 

### 3.1. Overview of SCI

Spinal circuits are susceptible to long-term neuromodulation that can alter how they function. Research studies from several laboratories including ours have focused on the types of neuromodulation (plasticity) of spinal networks that arise after SCI. Having established that BDNF functions as a neuromodulator of spinal networks, many studies examined reciprocal interactions between BDNF function and SCI and the consequence these interactions have on spinal cord networks and functions. SCI results in a myriad of behavioral and cellular consequences. Behaviorally, the effects of SCI include paralysis, muscle spasticity, and impaired bladder, bowel, and sexual function. Pain, including chronic neuropathic pain, is also a common debilitating consequence of SCI. The cellular changes induced by SCI, which typically drive these pathologies, include an increase in the proliferation and activation of glial cells, the release of proinflammatory cytokines from activated glia, glutamate spill, and excitotoxicity, which can eventually lead to necrotic and apoptotic cell death, for example, [106–108]. Secondary to these cellular effects are morphological changes such as afferent sprouting, cavitation, and gliosis-induced scarring. The milieu created by the collusion of these cellular and morphological changes has impeded the prospect of full repair after injury.

### 3.2. BDNF's Effect on Axonal Regrowth and Recovery of Locomotion after SCI

In the developing nervous system, BDNF plays an important role of promoting neuronal growth and survival. Thus, it was posited that similar effects of BDNF could be observed following SCI. Many studies have assessed whether BDNF can repair the injured spinal cord and rescue locomotor function after SCI. Schnell et al. [109] first investigated the effect of the neurotrophins, BDNF, and neurotrophin-3 (NT-3) on sprouting of corticospinal tract fibers after SCI. They found that whereas NT-3 promoted regeneration of these fibers, BDNF did not. Meanwhile, reports coming out of other laboratories reported neuroprotective effects of BDNF, especially after injury [110, 111]. Following these initial studies, there was an overwhelming increase in the number of research studies that focused on unraveling the neuroprotective effects of BDNF. Numerous studies examined whether treatment with BDNF could promote axonal regrowth across the injury site or sprouting of supraspinal projections. In many of these studies, approaches were implemented that allowed for successful long-term administration of BDNF, applications such as the use of mini osmotic pumps [112–114], or cellular grafts genetically modified to secrete BDNF [115–119]. Commonly, the results showed that BDNF treatment resulted in neuroprotection, as well as promotion of regeneration and sprouting of axonal fibers. Studies from the Houle laboratory showed BDNF-induced regeneration of descending supraspinal neurons, including serotonergic and vestibulospinal tract neurons, for example, [120–122]. Quite often it was also reported that behavioral recovery complemented the cellular effects. For example, BDNF enhanced the recovery of locomotor functions after SCI [113, 118]. Critical to BDNF's role in promoting axonal growth, afferent sprouting, and functional recovery, it was also shown in later studies that BDNF suppressed apoptosis in neurons and oligodendrocytes following SCI [123, 124].

### 3.3. BDNF Promotes Adaptive Plasticity after SCI

For many years the Grau laboratory has undertaken studies on spinal plasticity using a simple instrumental learning paradigm in adult rats with a complete thoracic (T) level 2 spinal transection. They showed that the induction and maintenance of spinal learning require functional NMDA receptors [125], reviewed by [126]. NMDA receptor dependence is a feature common to most types of plasticity such as learning and memory [127–130], synaptic plasticity (LTP and LTD) [129, 131, 132], and nociceptive plasticity [104, 133, 134]. The spinally mediated form of instrumental learning studied here similarly requires many of the cellular processes that are involved in other forms of plasticity such as kinase activity and protein synthesis [126]. As discussed, BDNF plays a role in the induction and expression of LTP. It can also induce synaptic facilitation and enhance excitability of spinal neurons, for example, [75, 89]. For these reasons, recent investigations focused on whether BDNF is equally involved in the processes that mediate isolated spinal learning. Using the standard Master-Yoke learning paradigm in T2 completely transected adult rats [135], cellular assays were undertaken to relate changes in the expression of BDNF and several “plasticity” genes with spinal learning [136]. For the Master-Yoke experimental design, rats are set up for instrumental learning in pairs. Each pair consists of one subject (Master), which is given response-contingent shock, wherein legshock is applied whenever the leg is extended and terminated when the leg is flexed (controllable shock). The second subject is experimentally yoked to the master rat and receives shock at the same time and for the same duration as the master rat but independent of leg position (uncontrollable shock, yoked). A third group, which serves as unshock controls, is set up in the same manner but does not receive any stimulation. Master-Yoke pairs and unshock controls undergo a 30-minute training phase. Following training, each subject undergoes instrumental testing for 30 minutes under the same conditions. During the testing phase, all three groups receive response-contingent shock. Immediately following instrumental testing, master rats that received controllable shock during the training phase and showed spinal learning during the testing phase had elevated spinal BDNF mRNA levels. In contrast, yoked subjects had decreased spinal levels of BDNF compared to unshock controls. Because yoked subjects fail to learn during the instrumental testing phase, these observations suggested that decreases in BDNF levels are detrimental to spinal learning [136].

**Figure 4:**
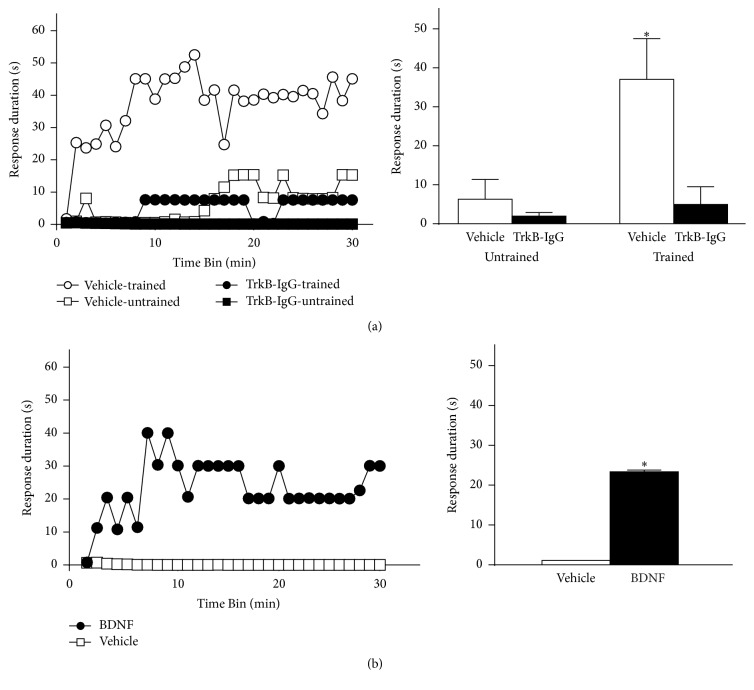
Role of BDNF in the protective effect of spinal instrumental training. (a) The necessity for endogenous BDNF in the spinal training effect. Rats received an intrathecal injection of either vehicle (saline) or the BDNF inhibitor TrkB-IgG and then either spinal instrumental training (trained) or none (untrained), followed by 6 min of uncontrollable shock. All subjects were then tested for instrumental learning over 30 minutes. Vehicle-treated subjects that were not trained prior to uncontrollable shock (vehicle-untrained) exhibited a learning deficit, whereas those prior training protected against this deficit (vehicle-trained). Subjects that received TrkB-IgG exhibited a learning deficit, regardless of training, indicating a necessary role for endogenous BDNF in the protective effect of spinal training. (b) To test the sufficiency for BDNF in the protective effect against uncontrollable shock, either vehicle or intrathecal BDNF was delivered prior to 6 minutes of uncontrollable shock. Rats that received vehicle prior to uncontrollable shock exhibited a deficit when tested for instrumental learning. Those treated with BDNF prior to uncontrollable shock were able to learn, indicating that BDNF is sufficient to protect against the maladaptive effects of uncontrollable shock (^*∗*^ *p* < 0.05). Error bars indicate SEM. Adapted from Huie et al. [91].

In a more recent study, we used* in situ hybridization* and western blot to assess mRNA and protein levels, respectively, following Master-Yoke training [91]. Elevated BDNF mRNA was observed in master subjects in both dorsal and ventral spinal cords. There was no difference in mRNA levels in unshock controls versus yoked subjects. Similar effects were observed with protein, in that BDNF levels were increased in master subjects but unchanged in yoked subjects compared to unshock controls. Additional experimentation provided further support that endogenous BDNF is critical to the protective effect of spinal training processes and that administration of BDNF prevents the spinal learning deficit induced by uncontrollable shock (Figure 4). Together, our prior results are consistent with those observed in other CNS regions, such as the hippocampus, where BDNF is explicitly shown to be involved in the neural processes that underlie learning [35, 137].

The role BDNF plays in spinal learning and other forms of plasticity after SCI is unequivocally linked to changes in the expression and function of the BDNF receptor, TrkB. In regard to the effect SCI has on TrkB expression within the spinal cord, conflicting reports exist although the differences may be due to temporal and spatial experimental variables. Specifically, TrkB expression after SCI typically depends on the postsurgical time points and spinal segments used for the cellular assessment. Frisén et al. [138] reported elevated levels of TrkB mRNA and protein expression in the spinal cord after SCI. Interestingly, the increases were most pronounced between 3 and 6 weeks after injury. However, in contrast, several other researchers have reported that TrkB levels are decreased by SCI [139–141], generally in the acute stage of injury. Interestingly, after T2 spinal transection rats that received controllable stimulation had increased protein expression of TrkB compared to yoked and control subjects [91]. Moreover, the increased TrkB expression predominated in the dorsal spinal cord, but not ventrally. It should be noted that although we found adaptive plasticity, that is, spinal learning, to associate with elevated levels of both BDNF and TrkB, we also observed decreases in BDNF and TrkB levels immediately after SCI (discussed below).

### 3.4. BDNF's Effect on Pain and Nociception after SCI

It would appear from these aforementioned studies detailing the progress made at the basic science level that the clinical use of BDNF after SCI is inevitable. Surprisingly, according to the U.S. National Institutes of Health (ClinicalTrials.gov), there is currently no account of clinical studies where BDNF is used to promote functional recovery after SCI even though BDNF is the focus of several non-SCI related clinical studies. The reasons for this apparent discrepancy and the slow progression of BDNF from “bench to bedside” despite the overwhelming promise shown at the basic research level are unknown but need to be explored in more depth. The following section discusses nociceptive plasticity after SCI and the differential effects of BDNF.

Like peripheral injury, central injuries including SCI produce chronic neuropathic pain [142, 143]. It is generally assumed that the mechanisms that underlie pain resulting from inflammation or peripheral injury are identical to those that underlie SCI-induced pain, with the main candidate being central sensitization [69]. Over the years, many investigators have investigated the mechanisms that produce central sensitization and identified key mediators (reviewed by [144]). Most notable are the NMDA receptors, increases in intracellular calcium, signaling kinases, and BDNF. More recently, additional mediators have been identified. These include the small molecular weight signaling proteins known as the cytokines and chemokine [145–147]. These signaling factors are generally released from immune-inflammatory cells. These recent discoveries add a new dimension to studying the development of pain hypersensitivity after SCI. No longer should we limit our attention primarily to neuronal plasticity, but rather we can also focus on the direct contributions of astroglial cells and the immune systems, as well as their ability to influence neuronal functions.

A hallmark feature of injury to the CNS is the subsequent accumulation, proliferation, and activation of glial cells. The observation that there is formation of a glial scar after injury to the CNS dates back to early reports by Cajal [148]. Moreover, it was shown then that the glial scar serves as a major inhibitor of neural regeneration. This early discovery was followed up by studies that focused on characterizing the cellular structure of the scar and evaluating possible means of overcoming this barrier to regeneration (for recent reviews, see [149–152]). Studies now indicate that the function of glial cells extends beyond the formation of a physical barrier after injury to the CNS. Both infiltrating immune cells and resident glia undergo structural and functional modifications after injury [153], ultimately enabling them to release several products. These include the inflammatory cytokines: interferon gamma, TNF alpha (TNF*α*) and the interleukins, and chemokines. Although they play various neural functions, many of these cytokines have been shown to promote deleterious effects after SCI, including the development of pain hypersensitivity and impaired locomotor functions. As reported by Coull et al. [96], activated microglia also release BDNF. Thus, one would expect that, together with the proinflammatory cytokines, the oversecretion of BDNF would only add to an environment that is favorable to maladaptive plasticity and pain hypersensitivity after SCI.

**Figure 5:**
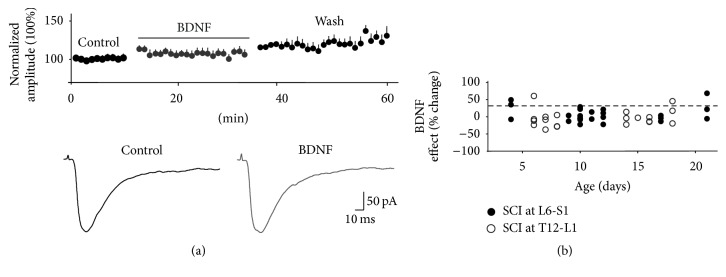
BDNF fails to induce synaptic facilitation after SCI. (a) Normalized data (mean ± SEM) averaged over all neurons show that synaptic facilitation was abolished after transection (at L6-S1 or T12-L1) or contusion (at T12-T13) SCI. (Some data points have smaller error bars than the points used to define the data.) Bottom: the average synaptic traces are shown for synaptic recordings obtained from a neonatal rat with contusion SCI before and during BDNF. (b) Mean percent change in synaptic amplitude in the presence of BDNF versus age is shown for each animal that received a transection injury. Although the effect of BDNF is somewhat variable, overall the effect is much reduced compared to the average facilitation of ~30% (represented by the horizontal dotted line) which was seen in uninjured animals of similar age. Also, BDNF's effects are not dependent on the location of injury. Recordings were done in L2–L5 lamina II neurons. From data previously reported in Garraway et al. [90, 92].

Despite the vast number of studies examining the behavioral effects of BDNF after SCI, few have looked at its cellular effects, especially in relation to nociceptive actions. This contrasts with the many studies aimed at understanding its role in neuropathic pain after peripheral nerve injury. Having shown that BDNF induces maintained facilitation of primary afferent-evoked synaptic responses in lamina II neurons in neonatal and young rats, an effect that is deemed pronociceptive [89], we turned our attention to investigate the actions of BDNF after SCI. Following a transection SCI at T13/L1 or L6/sacral 1 (S1) or a contusion SCI at T12/T13, BDNF did not facilitate afferent-evoked synaptic responses in L2–L5 lamina II neurons [90, 92] (Figure 5), which was an unexpected finding. Equally surprising was the observation that BDNF's failure to induce synaptic facilitation was not dependent on the type or location of SCI. It seemed plausible that BDNF would induce an even greater facilitation of synaptic responses after SCI. The expectation was based on the notion that if peripheral injury increases both spinal levels of BDNF and the excitability of lamina II neurons, a similar effect could be anticipated after injury to the spinal cord. Additionally, it was already shown that SCI increases spinal neuronal excitability or output, although in nonpain processing spinal regions [154, 155], that SCI results in increased proliferation and activation of microglia, and that activated microglia release of BDNF plays a crucial role in neuropathic pain [96]. In contrast, we found that failure of BDNF to produce synaptic facilitation was linked to an injury-induced increase in GABA_A_-mediated inhibition of spinal neurons coupled with suppression of NMDA receptor-mediated currents [92]. Although we did not observe any direct effect of BDNF on GABA currents, a previous study by Pezet et al. [156] showed that BDNF could directly stimulate the release of GABA from dorsal horn interneurons. Similarly, a study by Lu et al. [157] showed that, following long-term exposure of substantia gelatinosa neurons to BDNF, the amplitude of GABAergic inhibitory currents is enhanced. Together, these studies show a direct BDNF effect on GABAergic systems. However, it is unclear whether BDNF increases GABA levels after SCI.

The types of plasticity observed in developing nervous systems are not necessarily preserved into maturity. Therefore, before precise conclusions can be made on the kind of role BDNF plays in nociceptive plasticity after SCI, it is worthwhile to transition from a reduced preparation in juveniles to a mature, intact preparation which would allow for behavioral examination to be undertaken. We recently performed studies that investigated whether BDNF is involved in nociceptive plasticity and pain after SCI in an adult preparation, using the clinically relevant contusion SCI model at T12 [93]. The goal of the study was twofold: first, to assess the effect of SCI and noxious stimulation on the expression of key components of the BDNF signaling pathway and, second, to study interactions between the development of mechanical allodynia and changes in spinal expression levels of BDNF. Twenty-four hours after a moderate contusion injury, some adult rats received intermittent (~0.5 Hz) stimulation to the tail (SCI+SHK), at intensities that engage C fibers [158]. The remaining SCI subjects were placed in the shock apparatus but did not receive noxious tailshock (SCI+UNSHK). Our results first confirmed a prior study that showed that noxious tailshock negatively impacts the recovery of locomotion [159], as assessed by the Basso, Beattie, and Bresnahan (BBB) locomotor scale [160]. Our assessment of BDNF mRNA and protein levels showed that SCI alone significantly decreased both mRNA and protein levels at 25 hours and 48 hours after injury (equivalent to 1 hour and 24 hours after shock, resp.) (also see Strickland et al. [161]). However, noxious stimulation (SCI+SHK) produced an additional decrease in BDNF expression in the dorsal spinal cord. These changes in BDNF expression were nearly completely paralleled by TrkB mRNA and protein expression as well as that of the signaling kinases ERK1/ERK2 and CAMKII. In general, SCI decreased their expression levels during these early time points after injury, and noxious shock (SCI+SHK) produced additional decreases that were observed primarily in the dorsal spinal cord [93].

Our behavioral assessment showed that, during the acute postinjury period, when BDNF levels are reduced by SCI and noxious shock [93], the onset and magnitude of mechanical allodynia are significantly increased [94]. These observations suggest that neither the induction of nociceptive plasticity nor the onset of neuropathic pain after SCI is dependent on elevated levels of spinal BDNF after SCI. A similar finding was reported by Ferguson et al. [162] which indicated that, after transection SCI, intermittent noxious shock that decreased BDNF levels also increased mechanical reactivity to von Frey stimulation. Overall the results from these studies undertaken in adult rats with either a moderate contusion or transection SCI indicate the likelihood that short-term maladaptive plasticity after SCI is associated with deficits in spinal BDNF levels not increases.

**Figure 6:**
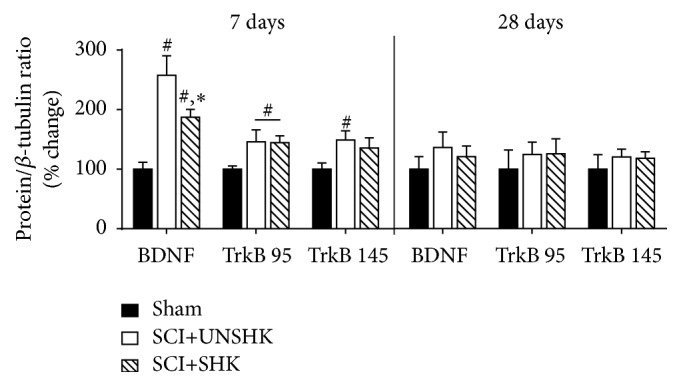
BDNF and TrkB expression in the dorsal spinal cord after SCI: Western blot was used to assess BDNF, TrkB_95_ and TrkB_145_ expression at 7 and 28 days after SCI in adult rats. Noxious tailshock was given 24 hours after SCI (SCI+SHK) to some animals. At 7 days after SCI, there was a significant increase in BDNF levels in the dorsal spinal cord of SCI animals that did not receive tailshock (SCI+UNSHK) and in SCI+SHK animals compared to sham controls. Although SCI+SHK animals had elevated levels of BDNF, shock treatment significantly decreased BDNF levels compared to SCI+UNSHK (^#^ *p* < 0.05 indicates significance relative to sham and ^*∗*^ *p* < 0.05 indicates significance relative to SCI+UNSHK). Both SCI groups had elevated levels of TrkB_95_ in the dorsal spinal cord compared to sham control, whereas TrkB_145_ was only significantly increased in the SCI+UNSHK group of animals. At 28 days after injury, there were no differences in BDNF, TrkB_95_, and TrkB_145_ expression in the dorsal spinal cord among the three experimental groups.

We extended the examination of the temporal changes in BDNF and TrkB expression in the dorsal spinal cord after SCI and noxious shock to 7 and 28 days after injury (Figure 6). The results revealed that SCI-induced reduction in BDNF and TrkB levels is transient. By 7 days following SCI, protein levels of BDNF, TrkB_95_, and TrkB_145_ were all elevated in SCI+UNSHK subjects compared to sham controls. Noxious stimulation produced a significant decrease in BDNF levels in the dorsal spinal cord, compared to SCI+UNSHK subject, although BDNF level remained elevated compared to sham controls. At 28 days after injury, BDNF levels and TrkB_95/145_ in the dorsal spinal cord were similar in all three groups. In fact, other studies have reported that TrkB levels are increased in the later stages of SCI [138, 163]. Overall, these data sets fully support the concept previously stated that spatial and temporal variables are important in determining the expression level of BDNF and TrkB after SCI.

### 3.5. Maladaptive Plasticity and Impaired BDNF Signaling

From these aforementioned studies, we can posit that SCI-induced maladaptive plasticity following SCI is in part mediated by impaired BDNF-TrkB signaling within the spinal cord dorsal horn [91, 93]. Previous studies from the Grau laboratory proposed that shock-induced behavioral deficits after SCI are mediated by spinal mechanisms that produce central sensitization [162]. However, the current discussion pinpoints a fundamental difference between the mechanisms that produce central sensitization after peripheral and central injuries. While central sensitization driven by BDNF increase is implicated in peripheral injury- and inflammation-induced pain, after SCI, central sensitization-like mechanisms that do not involve elevated levels of BDNF are likely to promote the induction/onset of chronic neuropathic pain [93]. Yet, whether BDNF potentiates maladaptive plasticity including the maintenance of neuropathic pain or other behavioral deficits incurred in the chronic phase of injury cannot be discounted by these observations. Actually, Boyce et al. [164] showed that, in the chronic stage of injury (6 weeks), contused rats treated with a viral vector expressing BDNF had enhanced sensitivity to heat stimulus (Hargreaves' test) compared to subjects treated with NT-3 expressing or control vectors. The idea that BDNF could produce pronociception in the chronic phase of injury is supported by the fact that the early reductions in BDNF and TrkB levels after SCI are reversed at later stages after injury, when maintained mechanical allodynia is evident [94]. A probable scenario is that BDNF exerts bidirectional functions following SCI. In the acute stage of injury, reduced levels of BDNF produce maladaptive spinal plasticity and impair behavioral function. Under those conditions, treatments that increase BDNF levels are beneficial. For example, elevated BDNF levels, whether from endogenous or exogenous source, have been shown to promote adaptive plasticity [91, 136, 165, 166] and functional recovery [164, 167, 168] after injury. However, in the more chronic time point when BDNF levels have been restored, overexpression of BDNF can again induce maladaptive plasticity which could in turn lead to pain hypersensitivity [94, 164]. Together, these observations highlight a potential limitation to the use of BDNF after SCI, the apparent shift from exerting adaptive effects to actions that are maladaptive. Moreover, because TrkB expression is also increased during the chronic stage of injury, even minimal overexpression of BDNF can initiate maladaptive plasticity.

Overall these studies signify tremendous ambiguity as it relates to BDNF functions following SCI. First, it is clear that there is divergence in the mechanisms that underlie central sensitization and pain hypersensitivity after SCI, such that it no longer requires BDNF-induced facilitation of synaptic efficacy. Although there are many overlaps in the underlying cellular and neural mechanisms that underlie inflammatory, peripheral injury, and neuropathic pain [169], trauma to the spinal cord appears to fundamentally alter these basic processes. Second, they show a distinction between how BDNF influences spinal nociceptive processes in intact and injured subjects and also reveal an important difference in how BDNF influences motor versus sensory systems after SCI. Undoubtedly, BDNF enhances adaptive plasticity and promotes recovery of locomotor function but appears to be less influential in directing nociceptive plasticity, although there is a possibility that BDNF activates pain systems at later stages of injury. It appears that while underexpression of BDNF can mitigate adaptive plasticity, overexpression appears to be equally detrimental. This has been demonstrated in the forebrain where overexpression of BDNF was found to be detrimental to memory and learning [170]. We believe that spinal nociceptive networks are similarly vulnerable to maladaptive changes if BDNF levels are abnormally enhanced after SCI.

## 4. Mechanisms Mediating BDNF's Effects

### 4.1. GABA Signaling Mechanisms

As previously mentioned, a complex interaction between BDNF and GABA appears to play a pivotal role in neuropathic pain after peripheral injury [96]. Lu et al. [99] showed that, in young rats following sciatic nerve injury, BDNF alters the electrophysiological properties of dorsal horn neurons. Specifically, BDNF increases both the excitatory and inhibitory synaptic drives to putative excitatory interneurons while attenuating synaptic transmission to inhibitory GABAergic neurons.

**Figure 7:**
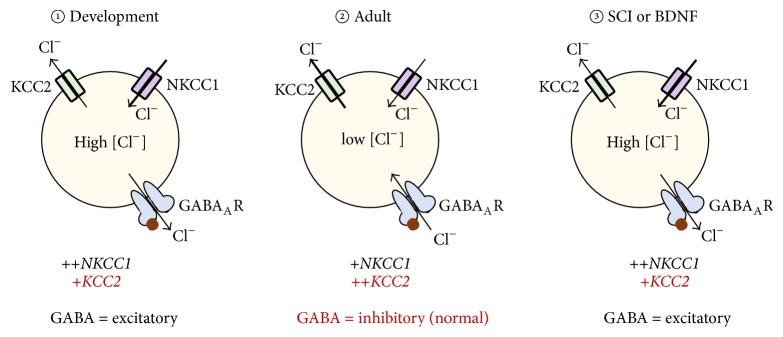
Plasticity in GABA-mediated chloride function; role of chloride transporters KCC2 and NKCC1. ① During development, CNS neurons express high levels of NKCC1 and low levels of KCC2. Upon GABA binding GABA_A_ receptors, chloride ions [Cl^−^] exit the cell, thereby producing excitatory actions. ② In mature cells, the reverse occurs. The high concentration of KCC2 causes [Cl^−^] to enter the cell, which results in GABAergic inhibitory actions. ③ Both SCI and BDNF have been shown to decrease membrane bound KCC2 expression on neurons. This effect causes a shift in GABA function from inhibition to produce excitatory effects. This switch may contribute to pain after SCI.

The multifaceted BDNF-GABA relationship after peripheral and spinal cord injury is directly linked to the ability of both BDNF and SCI to independently regulate the expression and function of chloride transporters. K^+^Cl^−^ cotransporter 2 (KCC2) plays an important role in regulating neuronal chloride homeostasis and is primarily responsible for the inhibitory actions of GABA [171, 172], unlike its depolarizing counterpart Na^+^K^+^Cl^−^ cotransporter 1 (NKCC1) [173, 174] (Figure 7). In immature neurons where intracellular concentration of chloride ions [Cl^−^]_i_ is high, NKCC1 is highly expressed, thereby mediating the excitatory action of GABA seen during early development. However, in adult neurons, the inhibitory actions of GABA are due to a predominant KCC2 expression. GABA receptor functions and chloride homeostasis are influenced by manipulations that modify the expression of NKCC1 and KCC2. In hippocampal cells, BDNF decreased both the expression and phosphorylation of KCC2 [175, 176] and, in the nucleus raphe magnus, KCC2 levels were decreased by BDNF, producing an overall excitatory result that is mimicked by inflammatory pain [177]. This latter study suggests that, at supraspinal pain processing center, BDNF can regulate chloride homeostasis. It further implies that persistent pain resulting from decreased expression of KCC2 can be induced by BDNF's administration.

Nonetheless, different observations are noted after SCI. Boulenguez et al. [178] recently showed that SCI decreases the protein expression of KCC2 in the spinal cord (also see [179]), the same effect produced by BDNF. When BDNF was administered to spinal cord of intact subjects, there was a reduction in plasmalemmal KCC2 expression which is the converse of the increase caused by BDNF 15 days after SCI. Thus, it appears that BDNF, like SCI, can decrease KCC2, whereas BDNF plus SCI causes an increase in KCC2. Boulenguez et al. [178] also showed that the early decrease in KCC2 caused by SCI could be prevented by inhibiting endogenous BDNF with TrkB-IgG, which suggests that SCI-induced decrease in KCC2 is mediated by BDNF. Studies by Cramer et al. [179] and Hasbargen et al. [180] also reported that SCI dramatically reduces KCC2 but increases NKCC1 levels. When the results from these independent studies are collated, there is succinct evidence that BDNF-induced plasticity is highly susceptible to SCI-induced modification and* vice versa*. Therefore, it can be surmised from these studies that because a decrease in KCC2 expression is consistent with increase excitability, then BDNF exerts opposing actions in intact and injured spinal cord. Specifically, while it reduces KCC2 which promotes excitation (pronociceptive effects) in intact spinal cord, after SCI, BDNF could produce protective or antinociceptive effects by increasing KCC2 and GABAergic inhibition. Clearly, there are no solid explanations for these differential effects of BDNF or the mechanisms that trigger a tangible switch in BDNF functions after SCI. Both small diameter primary afferents and activated microglia are likely sources of increased levels of BDNF within the spinal cord. If high levels of BDNF are retained into the chronic stages of injury presumably after the primary injury has been healed, then probable mechanism of maintained pain hypersensitivity would be BDNF-induced reduction in KCC2 mediated GABAergic inhibition. However, although the effect BDNF has on KCC2 and GABA circuits in the chronic stage of injury is clearly speculative at this time, it seems that SCI-induced reduction in KCC2 expression is considered a possible mechanism that underlies acute or early onset pain after SCI.

### 4.2. ERK-pERK Signaling

**Figure 8:**
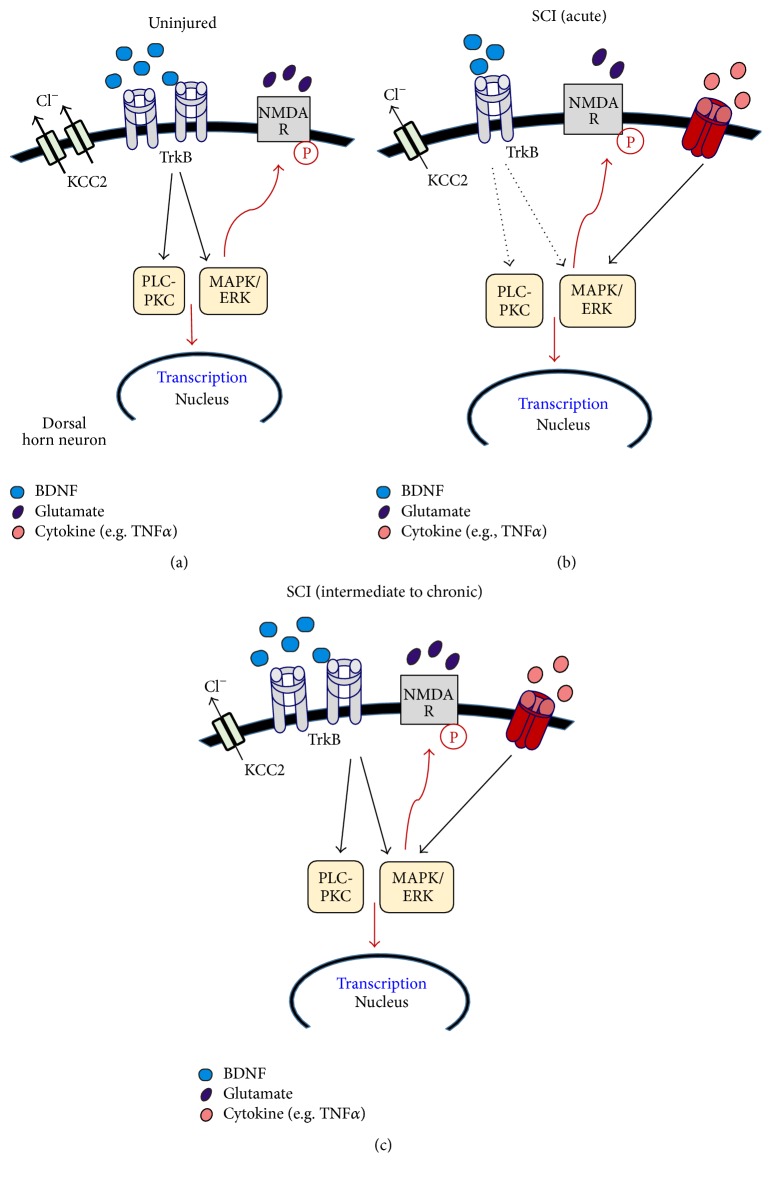
Potential BDNF-dependent and BDNF-independent mechanisms of pain in uninjured and injured spinal cord. (a) In the absence of SCI, BDNF-mediated effects contribute to pain. Constitutive increases in BDNF and TrkB expression activate MAPK/ERK and PLC-PKC kinase pathways, which in turn leads to transcription of pain genes and posttranslational modifications (e.g., phosphorylation of glutamate receptors). (b) In the acute stage of SCI, both BDNF and TrkB levels are decreased in the spinal cord dorsal horn [93]. However, during this stage, MAPK/ERK pathways could be activated by BDNF-independent mechanisms, presumably an alternate pathway such as the TNF*α* pathway (both TNF*α* and TNR1 expressions are upregulated by SCI [94]). A decrease in KCC2 by SCI which alters GABA-mediated chloride function is likely to also contribute to pain after SCI. (c) During the chronic stage of injury, pERK levels are increased [94, 95], which may result from increases in both BDNF-TrkB signaling and TNF*α*-TNFR signaling. Activation of these pathways could consequently increase kinase activity and the transition of pain genes. Overall, we propose that although BDNF may not necessarily initiate pain-producing pathway after SCI, it is likely to contribute to pain hypersensitivity during the chronic stages of injury.

As we discussed earlier, ERK-dependent signaling mechanisms are involved in the development of pain. Interestingly, in our studies, we observed a decrease in ERK1/ERK2 and activated ERK1 (pERK1) expression at the lesioned epicenter during the first 7 days after SCI [93] although a robust increase in pERK1/pERK2 was observed in the ventral spinal cord at 28 days after SCI, at a time when rats showed mechanical allodynia [94]. Crown et al. [95] reported an increase in pERK1/pERK2 in the spinal cord of SCI rats expressing neuropathic pain behaviors at 35 days after injury. Together, these studies suggest that, after SCI, ERK expression and phosphorylation correlate to the development of pain and this is not merely due to the SCI* per se.* It is not determined whether these changes in ERK expression are determined by BDNF-TrkB signaling or whether BDNF-induced activation of these neural substrates plays an important role in producing neuropathic pain after SCI particularly as ERK can be engaged by other signaling pathways such as TNF*α*. Moreover, as we and others have shown, both TNF*α* and its receptors (TNFR1 and TNFR2) are upregulated after SCI [94] and may contribute to chronic pain states after SCI. In Figure 8, we present a brief summary of these possible mechanisms of pain in uninjured and injured spinal cord.

Importantly, it should be noted that although SCI appears to mimic some of BDNF's detrimental actions, these effects may not be mediated by BDNF itself. The phenomenon termed “metaplasticity” by Abraham and Bear [181] is a change in the ability to induce subsequent synaptic plasticity. The fact that SCI impacts the manner and type of BDNF-induced plasticity particularly as it relates to the nociception and pain after SCI is an important example of spinally mediated metaplasticity. While additional experiments are needed to completely resolve this issue, the simplest conclusion to be made from these studies is that the use of BDNF following SCI does not present a challenge to spinal nociceptive and pain processing, at least acutely.

## 5. Conclusion

Studies of spinal plasticity have revealed that the processes involved are indeed complex. An even greater level of complexity arises after SCI, partly due to the enormous amount of cellular and morphological changes that ensue. BDNF has been classified as a neuromodulator and has been shown to be intricately involved in spinal plasticity. Clearly, there is no uniformity with BDNF actions as it has been shown that it can mediate both adaptive plasticity and maladaptive plasticity in spinal networks. For many years, our studies focused on unraveling these differential effects BDNF has on spinal networks in uninjured and injured spinal cord. Deductions made from several studies including some from our laboratories overwhelming show that as it relates to nociceptive processes, whereas BDNF exerts pronociceptive actions in the absence of injury, no such effect is observed after injury, at least acutely. Moreover, we found that functional deficits and mechanical sensitivity after SCI are associated with reduced BDNF levels, not increases. In contrast, increases in BDNF after SCI promote adaptive plasticity and functional recovery. The multifaceted effects of BDNF after SCI may be linked to SCI-induced changes in (i) expression of TrkB receptor and downstream kinases, (ii) GABA transmission due to altered expression of chloride transporters, and (iii) the activity of astroglial cells. The idea that BDNF plasticity is potentially susceptible to SCI-induced modifications indicates that metaplastic changes are essential to spinal function, particularly after injury to the CNS. In conclusion, we show that while significant progress has been made towards a clearer understanding of BDNF functions, after 30 years, additional research is warranted to elucidate the spinal effects of BDNF-TrkB signaling.
